# Comprehensive survey on the use of plastic additives in toy products used in Japan

**DOI:** 10.1265/ehpm.24-00054

**Published:** 2024-08-22

**Authors:** Kanae Bekki, Akifumi Eguchi, Kohki Takaguchi, Yohei Inaba, Keiko Yukawa, Satomi Yoshida, Kenichi Azuma

**Affiliations:** 1 Department of Environmental Health, National Institute of Public Health, 2-3-6 Minami, Wako-shi, Saitama 351-0197, Japan; 2 Center for Preventive Medical Sciences, Chiba University, 1-33 Yayoi-cho, Inage-ku, Chiba-shi, Chiba 263-8522, Japan; 3 Department of Health Policy and Technology Assessment, National Institute of Public Health, 2-3-6 Minami, Wako-shi, Saitama 351-0197, Japan; 4 Department of Pharmacoepidemiology, Graduate School of Medicine and Public Health, Kyoto University, Yoshidakonoecho, Sakyo-ku, Kyoto 606-8501, Japan; 5 Department of Environmental Medicine and Behavioral Science, Kindai University Faculty of Medicine, Ohnohigashi Osakasayama, Osaka 589-0014, Japan

**Keywords:** Nontargeted analysis, Toy, Phthalate ester, Phosphorus flame retardant

## Abstract

**Background:**

Plastic additives have adverse effects on human health. Children frequently use toys that contain various substances found in paints, plasticizers, and other materials, which heighten the risk of specific chemical exposure. Infants are particularly prone to chemical exposure through the “mouthing” behavior because of the possibility of placing toys in their mouths. Thus, this vulnerability should be considered during risk assessments of chemical exposure.

**Methods:**

This study performed a comprehensive analysis of the chemical components in various 84 plastic toys including “designated toys” (toys that may be harmful to infant health if in contact with their mouths: Article 78 of the Enforcement Regulations of the Food Sanitation Law by the Minister of Health, Labor and Welfare) such as dolls, balls, blocks, bathing toys, toy vehicles, pacifiers, and household items, purchased in the Japanese market by nontargeted and targeted analysis.

**Results:**

Plasticizers, flame retardants, and fragrances were the main compounds in almost all the toy products. The results showed that plastic products made in China tended to contain high levels of phthalate esters. In particular, hazardous plasticizers, such as diisodecyl, di-n-octyl, and diisononyl phthalates were detected above the regulatory limit (0.1%) in used products manufactured before regulations were passed in Japan. Furthermore, we detected alternative plasticizers, such as acetyl tributyl citrate (ATBC; 52%), diisononyl adipate (DINA; 50%), and di(2-ethylhexyl) terephthalate (DEHT; 40%). ATBC was detected at high concentrations in numerous toy products. Thus, infants with free access to indoor plastic toys might be exposed to these chemicals.

**Conclusions:**

This study observed that the chemical profiles of toy products were dependent on the year of manufacture. Furthermore, the detection of currently regulated plasticizers in secondhand products manufactured before regulations were enforced, along with the increasing trend of using alternative substances to regulated phthalate esters in products, suggests the potential exposure of infants to these plasticizers through the use of toys. Therefore, regular fact-finding surveys should continue to be conducted for the risk assessment and safety management of domestic toy products.

**Supplementary information:**

The online version contains supplementary material available at https://doi.org/10.1265/ehpm.24-00054.

## Graphical Abstract

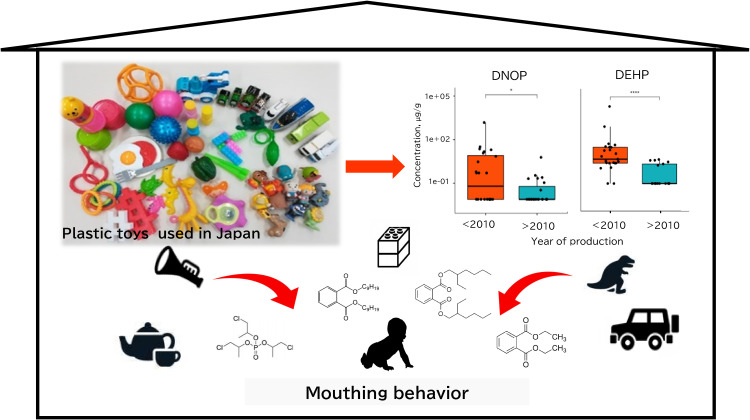

## Introduction

Recently, efforts have been devoted to analyzing the impact of chemical exposure on the growth and health of children [1]. Children frequently use toys that contain various substances found in paints, plasticizers, and other materials, which heighten the risk of specific chemical exposure [2–4]. Infants are particularly prone to chemical exposure through the “mouthing” behavior because of the possibility of placing toys in their mouths. Thus, this vulnerability should be considered during risk assessments of chemical exposure [5].

For example, in Japan, regulated substances in toy products mainly include heavy metals, such as lead, arsenic, and cadmium, along with phthalate esters (PAEs) used as coloring agents and plasticizers. PAEs can have adverse health effects, such as reproductive toxicity when ingested, due to their environmental hormone-like properties [6] and allergic reactions [7]. Therefore, they have become a focus of regulatory efforts in developed countries. In Japan, since 2010, the use of more than 0.1 wt% of dibutyl phthalate (DBP), di(2-ethylhexyl) phthalate (DEHP), and butylbenzyl phthalate (BBP) in plasticized materials for toys for children under 6 years old is prohibited (designated toys; Article 78 of the Enforcement Regulations of the Food Sanitation Law). In addition, the use of over 0.1 wt% of diisodecyl phthalate (DIDP), diisononyl phthalate (DINP), and di-n-octyl phthalate (DNOP) in the plasticized components of items, such as pacifiers and toothpicks, for infants to put in their mouths, has been banned. The use of DINP in toy products for infants to put in their mouths is also restricted. Following these regulations, toy products sold in the domestic market undergo safety inspections through testing methods compliant with Food Sanitation Law standards. However, recently, the use of alternative substances with similar structures (di(2-ethylhexyl) terephthalate (DEHT), acetyl tributyl citrate (ATBC), etc.) and substitutes for PAEs has increased. And, phosphorus flame retardants (PFRs) are increasingly used in products as brominated flame retardant (BFR) substitutes and have been linked to several health conditions, such as allergies, cancer, and nervous system challenges [8, 9]. In addition, used products may not be subject to regulatory oversight, implying that products containing regulated components could be circulating in the market. Furthermore, certain plasticizers can migrate from the product to the environment through, for instance, indoor dust, which has been reported to have potential health risks [10, 11]. In addition, the wide range of available household items has significantly transformed how infants and toddlers engage with toys, leading to a substantial increase in the use of tablet-based products. Thus, children, particularly infants and toddlers who place plastic toys in their mouths and frequently handle them, could be potentially exposed to hazardous plasticizers.

Therefore, in this study, we try to comprehensively investigate the use of regulated PAEs, their substitutes, and other plastic additives in domestic toy products to obtain data for risk assessments and the safety control of hazardous plasticizers for future regulations.

## Materials and methods

### Chemicals and reagents

Nine PAEs, including six compounds regulated in Japan, seven alternative plasticizers, and 14 PFRs were targeted. Dimethyl phthalate (DMP), diethyl phthalate (DEP), ATBC, and dibutyl sebacate (DBSb) were purchased from Tokyo Chemical Industry (Tokyo, Japan). In addition, 1,2-cyclohexane dicarboxylic acid diisononyl ester (DINCH) was purchased from BLDpharm (Shanghai, China). Diisononyl adipate (DINA), DEP-*d_4_*, DMP-*d_4_*, BBP-*d_4_*, DEHP-*d_4_*, DBP-*d_4_*, and DNOP-*d_4_* were purchased from Fujifilm Wako Pure Chemical Corporation (Osaka, Japan). DBP, BBP, DEHP, DNOP, DIDP, diisobutyl phthalate (DIBP), dicyclohexyl phthalate (DCHP), and DINP were purchased from Kanto Chemical Corporation Incorporated (Tokyo, Japan). Bis-(2-ethylhexyl) adipate (DEHA), acetonitrile, and ammonium acetate (≥99.99%) were purchased from Sigma–Aldrich Inc. (St. Louis, MO, USA). Trimethyl phosphate (TMP), triethyl phosphate (TEP), tris(2-butoxyethyl) phosphate (TBOEP), tris(2-chloroethyl) phosphate (TCEP), tris(1,3-dichloroisopropyl) phosphate (TDCIPP), triphenyl phosphate (TPHP), tricresyl phosphate (TCsP), 2-ethylhexyldiphenyl phosphate (EHDPhP), and cresyl diphenyl phosphate (CsDPhP) were purchased from Tokyo Chemical Industry (Tokyo, Japan). Tripropyl phosphate (TPP), tris(isobutyl) phosphate (TIBP), tris(2-chloroisopropyl) phosphate (TCIPP), and methanol were purchased from Fujifilm Wako Pure Chemical Corporation (Osaka, Japan). TCEP-*d_12_*, TPHP-*d_15_*, tris(methylphenyl) phosphate-*d_21_* (TMPP-*d_21_*), and TEHP-*d_51_* were purchased from Hayashi Pure Chemical Industry (Osaka, Japan). Water was obtained from a Milli-Q water purification system (Millipore, Bedford, MA, USA).

### Sampling of toy products

Toy products were obtained in the market based on statistical data from manufacturers of baby goods, the Japan Toy Association, Rakuten. baby toy rankings etc. [12, 13]. Among listed toy category in the data of the Japan toy association, 84 toy products, including “designated toys” (toys that may be harmful to infant health if in contact with their mouths: Article 78 of the Enforcement Regulations of the Food Sanitation Law by the Minister of Health, Labor and Welfare), were purchased via the internet or from toy stores, supermarkets, and dealers of second-hand goods in Saitama prefecture. At this time, we used the manufacturing date information indicated on the products and packaging, as well as the manufacturing date information available on the toy manufacturers’ websites, as one of the criteria for selecting products. These toy products were manufactured around 2010, when local regulations were expanded, and the products were manufactured from plastics made of various synthetic resins, such as polyvinyl chloride (PVC), polyethylene (PE), acrylonitrile butadiene styrene (ABS), and thermoplastic elastomer (TPE) (Table S1). Of these, 69 and 15 products were designated and undesignated toys, respectively. The designated toys included 15 dolls, 2 balls, 3 blocks, 2 bath toys, 11 vehicle toys, 9 pacifiers, 23 baby toys, and 4 other toys (Table 1). The undesignated toy products included puzzles, beads, and smartphone cases. Details of these products are shown in Table S1, along with photos of the products in Fig. S1.

**Table 1:** Number of designated and undesignated toys.

**Sample**	**Toys for ages 6 and under** **(Designated toys)**	**Others** **(Undesignated toys)**	**Total**
Doll	15	1	16
Ball	2	2	4
Block	3	0	3
Bathing toy	2	0	2
Toy vehicles	11	4	15
Pacifier	9	0	9
Play house toys	23	0	23
Others	4	8	12
Total	69	15	84

### Sample pretreatment

To pretreat each toy sample, after all the products contacting parts intended for the mouths of infants were cut and shredded with a plastic cutter, they were pulverized using a freezing pulverizer (JFC-400 freezing pulverizer, Japan Analytical Industry Co.). These samples were hermetically sealed in containers and stored in a cool, dark place at room temperature until analysis. For pretreatment, the crushed sample (50 mg) was sonicated in acetonitrile (5 mL, 40 °C, 40 min). The fraction (1 mL) was cleaned up using a solid phase extraction column (Bond Elut C18, Agilent) and concentrated under nitrogen flow. In this procedure, the same pretreatment was also performed for the blank control without the toy sample, and the value of the blank control was subtracted from the result of the toy sample to eliminate the contamination. Finally, an internal standard was added to the sample, which was filtered (0.2-µm pore size) prior to analysis. These pretreatment methods are based on the Food Sanitation Test Methods [14] and were newly devised to optimize conditions for the pretreatment and LC-MS/MS analysis of flame retardants, plasticizers, and alternative plasticizers in plastic products.

### Nontargeted and targeted analytical methods

A nontargeted analysis was performed via high-performance liquid chromatography quadrupole time-of-flight mass spectrometry (LC-QToFMS) (X500R, Sciex) using a Sciex Exion LC AD (X500R). An Ascentis Express C18 column (100 mm × 2.1 mm, 2.7 µm, Phenomenex) was used with 0.1% formic acid solution (solution A) and 0.1% acetonitrile solution (solution B) as mobile phases under the gradient conditions presented in Table S2-1. Peak detection and alignment were performed using MS-DIAL ver. 4.8.0 [15]. In addition, peak annotation was performed using MS-DIAL 4 with an in-house mass spectral library, including NIST20, Massbank of North America, human metabolome database, and MS-DIAL metabolomics MSP spectral kit. The annotated peaks that satisfied the Metabolomics Standards Initiative (MSI) Level 2 grade criteria for the toy sample analysis of MS and MS/MS spectral agreement via high-resolution MS were included in the analysis [16]. We linked the compounds with annotations to the CompTox Chemicals Dashboard database using InChIKey, and only those that existed in the database were targeted for data analysis [17]. Furthermore, compounds with detection rates below 20% were excluded from the analysis.

Target PAEs and alternative plasticizers were analyzed by highly sensitive and selective liquid chromatography-tandem mass spectrometry (LC-MS/MS) apparatus ACQUITY UPLC I-Class/Xevo TQ-S IVD System (Waters, MA, USA) in the multiple reaction monitoring (MRM) mode (Table S3-1) under the gradient conditions presented in Table S2-2. DEHP and DEHT, whose overlapping peaks were observed, were analyzed by gas chromatography-mass spectrometry (GC-MS) (QP2010 Plus) (Shimadzu, Kyoto, Japan) (Table S2-3) and quantified in the selected ion monitoring (SIM) mode. Target PFRs were analyzed by LC-MS/MS (Waters, MA, USA) in the MRM mode (Table S3-2), and the gradient conditions are listed in Table S2-4.

### Calibration curve and method validation

To establish a quality control protocol for this method, calibration curves were drawn from the ratios of the areas of the peaks due to six known concentrations of the PAEs, alternative substances, PFRs, and internal standards.

The limit of detection (LOD) and limit of quantification (LOQ) for the measurement of each plasticizer and flame retardant were defined as three and ten times the standard deviation (SD) of the lowest standard, respectively. To measure the accuracy and precision of the analytical method, the toy samples were spiked with standards of each plasticizer and flame retardant until a final concentration of the standard was reached (100 ng/mL). The accuracy was calculated from the ratio of the quantified concentration to the known concentration of the spiked analytes, whereas the precision was calculated from the relative SD (RSD, %) of the replicate.

### Statistical analysis

The data obtained via the nontargeted analysis was subjected to sparse principal component analysis (PCA) using the statistical analysis software, R ver. 4.1.2, FactoMineR [18], and mixOmics package [19].

## Results and discussion

### Comprehensive nontargeted analysis of plastic additives in toys

**Fig. 1:**
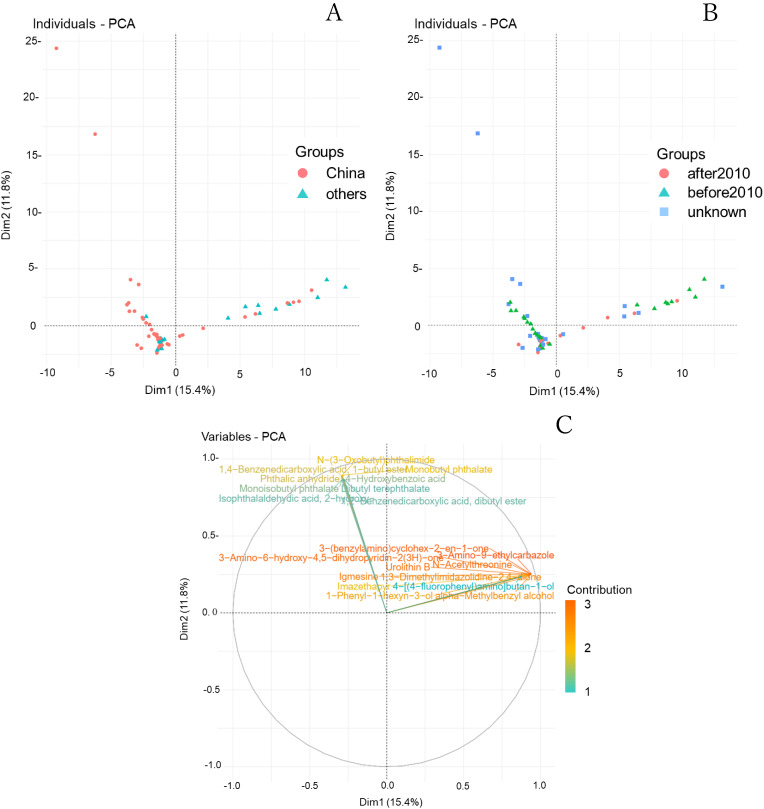
PCA score plot for comparison of countries of manufacture (A), comparison of year of manufacture (B) and PCA loading plot from Comprehensive analysis of plastic toys by LC-QToFMS/LC-MS/MS (C). The compounds shown in Fig. 1C are the top 20 contributing components in the principal component analysis.

Many chemicals are used as additives in plastic products to prevent degradation and enhance flexibility. Their use is continually rising because of the increased demand for alternatives to regulated ingredients. As these additives are highly variable, we employed LC-QToFMS to conduct a comprehensive nontargeted analysis of toy products widely used in Japan to determine the potential risks. First, we identified about 100 substances in the toy samples and observed numerous plasticizers, flame retardants, fragrances, coloring agent, etc. (Table 5S). We used the peak intensities to perform PCA to examine the distribution of the components according to the year of manufacture and country of production. Thus, the almost samples were classified into products made in China and those made in other countries (Fig. 1A). Similarly, principal component analysis of products made before and after 2010 showed classification trends similar to those observed in Fig. 1A (Fig. 1B). These results indicated that many samples made in China before 2010 were plotted in the second quadrant, and samples made in China which were not identified manufacturing dates were also distributed in the second quadrant, suggesting that these products also might have been manufactured before 2010. Furthermore, the compounds shown in Fig. 1C are the top 20 contributing components in the principal component analysis. This second quadrant primarily contained substances associated with health effects, such as allergic reactions and reproductive toxicity, including monobutyl phthalate and monoisobutyl phthalate [20, 21] (Fig. 1C). On the other hand, first quadrant included flavor, amino acid derivative, coloring agent etc. such as urolithin B, n-acetylthreonine, 3-amino-9-ethylcarbazole etc. Some of these ingredients appeared to be common in the base materials for plastics, but some of these also had uncertain origins and potential harmfulness. For these substances, it is necessary to thoroughly investigate the content within the product and the potential for leaching from the product in order to evaluate the potential risks as a future study.

From these results, it is suggested that products manufactured before 2010 contained relatively high amounts of harmful compounds, and the difference in the country of manufacture may also influence the composition of these ingredients in the toy products. Furthermore, since products manufactured after 2010 also contain substances that may indicate harmfulness, further research is necessary to comprehensively evaluate the health risks of these ingredients.

### Calibration curve and method validation

To determine the concentration of PAEs and alternative substances, we drew up calibration curves based on the concentrations of each standard solution with the extract obtained in the presence of the toy sample by pretreatment using a solid phase extraction column. Each calibration curve exhibited good linearity (Table S4-1). The calibration curves of the PFRs also exhibited good linearity (Table S4-2).

The recovery rates of all the PAEs and alternative substances were in the range of 80–123% respectively, except for DCHP (73%) and DMP (142%). The RSDs, measures of the precision of the method, were in the range of 0.9–4.9%, except for DMP, which was characterized by an inter-day RSD of 18.9%. The recovery rates of all the PFRs were in the range of 85–111% respectively, except for TDCPP (63%), TBOEP (62%), and EHDPP (69%). The RSDs, measures of the precision of the method, were in the range of 0.5–15%.

### Targeted analysis of PAEs and alternative substances in plastic toys

Based on the results of the nontargeted analysis, several PAEs which were concerned to induce health effects were detected in the toy products. To obtain more information, we conducted a targeted analysis of PAEs, and their concentration and substitutes in toy products are listed in Table 2. Among the components detected in the toys for children aged six and under, the average values of three substances were below the regulatory limit (<0.1 wt%). The concentrations of regulated PAEs in this study were lower than those reported by Sugita et al. [22] in the 1990s. The aforementioned previous study observed high concentrations of regulated PAEs, such as DINP (380–580 mg/g) and DBP and DEHP (3.0–100 mg/g) in products, such as pacifiers and soft toys, for children aged six years and under.

**Table 2:** The content rate of plasticizers and other materials in designated and undesignated toys (wt%).

	**Compounds**	**Toys for ages 6 and under (Designated toy) (n = 69)**			**Others (Undesignated toy) (n = 15)**		
	
		**Minimum**	**Average**	**Maximum**	**Minimum**	**Average**	**Maximum**
Regulatory Compounds	DBP	<0.1	0.73	1.9	<0.1	<0.1	<0.1
	BBP	<0.1	<0.1	<0.1	<0.1	<0.1	<0.1
	DEHP	<0.1	<0.1	2.5	<0.1	<0.1	<0.1
	DNOP	<0.1	<0.1	3.1	<0.1	<0.1	<0.1
	DINP	<0.1	0.88	6.6	0.19	1.8	5.6
	DIDP	<0.1	5.5	62	0.57	0.72	0.87

Alternative compounds	DEHT	<0.1	6.1	37	<0.1	<0.1	<0.1
	DIBP	<0.1	2.0	7.1	<0.1	<0.1	0.27
	DMP	<0.1	0.17	0.23	<0.1	<0.1	0.36
	DEP	<0.1	0.29	0.90	<0.1	<0.1	0.21
	DCHP	<0.1	<0.1	<0.1	<0.1	<0.1	<0.1
	DEHA	<0.1	1.8	7.6	<0.1	<0.1	<0.1
	DINA	<0.1	3.7	24	0.10	0.44	0.88
	ATBC	<0.1	9.9	61	0.10	12	48
	DINCH	<0.1	1.7	7.4	<0.1	<0.1	<0.1
	DBSb	<0.1	2.5	16	<0.1	<0.1	<0.1

<0.1: Content rate of regulatory and alternative compounds was under the regulated value of 0.1%.

On the other hand, finger puppet toy products in this study contained up to 62% DIDP, which was presumed to be a raw material used in manufacturing. Thus, products in which DIDP was used as a raw material accounted for 1.1% of the total. However, the DIDP in this product was not subject to regulations because this product was not initially intended to come in contact with the mouths of infants (Article 78-1 of the Food Sanitation Law Enforcement Regulations). Moreover, this product was relatively old, manufactured in the 1990s, and purchased from a dealer of second-hand goods, suggesting that it was manufactured or imported before the six PAEs were regulated in Japan. Similarly, toys containing over 0.1 wt% of DNOP and DINP were not targeted by the regulations. However, as these plasticizers have actually been detected in toy products that might be used daily and may adhere to toys through other household products, daily use of secondhand toys or household products containing regulated substances could result in exposure to harmful plasticizers. Thus, the safety management of used products is crucial. Furthermore, toys for children aged 6 and under exhibited a high detection rate of ingredients other than regulated PAEs, including DINCH (95%), ATBC (51%), DEHT (39%), DINA (52%), and DIBP (79%). The content of each ingredient in the products was as follows: DINCH (<0.1–7.4 wt%), ATBC (<0.1–6 wt%), DEHT (<0.1–37 wt%), DINA (<0.1–24 wt%), and DIBP (<0.1%–7.1 wt%). These results revealed the substitution of regulated PAEs for alternative plasticizers and a clear effect of the regulations and safety control for PAEs in toy products. Based on the results of the present survey, there were fewer deviations from the regulations for designated toys handled in Japan. Furthermore, the effects of safety management in Japan were more evident than before the drastic regulations of PAEs in 2010. Furthermore, Abe et al. (2019) [23] reported that although PAEs did not exceed the regulation values in the designated toys, alternative ingredients, such as DEHT, DINA, and ATBC, were relatively high. These results were consistent with the present study. Regulated PAEs and other substitutes were detected in recycled products in the present study, some of these products are used daily in households and public places. Despite these risks, there is no management system for local and international products in our country. This necessitates periodic substantive surveys of domestic toy products in the market for health risk assessments and safety evaluations for domestic use.

“Undesignated toys” including smartphone cases, beads, and balls for children aged six and older contained regulated PAEs and various other substitutes. Although we detected regulated PAEs, such as DINP (62%) and DIDP (15%) in the samples, they were present in only small amounts (0.19–5.6 and 0.57–0.87 wt%, respectively). In addition, trace amounts of alternative ingredients, such as DEP (<0.1–0.21 wt%), DINA (<0.1–0.51 wt%), and ATBC (<0.1–0.3 wt%), were detected in the smartphone case samples. In recent years, many infants and toddlers have started using new products such as smartphones, which may expose them to harmful chemicals from toys and household items. Regarding alternative substances in other products, the content and detection rates of DINA and ATBC tended to be relatively high compared with those of other alternative plasticizers (Table 2). These results showed that regulated PAEs in undesignated toys had been replaced by DINA and ATBC. In particular, ATBC was detected in PVC products at high concentrations (Fig. S5) and constituted nearly half (48 wt%) of the chemicals in PVC balls. A similar trend was reported (ATBC; 50 wt% (2009), 29 wt% (2014)) by Abe et al. in Japan. These data show that ATBC continues to be used as a major alternative in PVC products. However, the toxicity information of ATBC might be unknown, which necessitates a detailed toxicity assessment for this plasticizer. As risk assessments in previous studies conducted for PAEs mainly focused on PVC products, we focused on various plasticizers and substitutes used in products made of other plastic materials. Thus, official analytical methods and surveys for plastic products other than PVC materials are also required.

### Targeted analysis of PFRs in plastic toys

We measured 14 PFRs widely encountered in daily life. Of these, six PFRs were detected in certain products at a detection rate of below 50%. The detection rate of TPHP (15%) was relatively high, whereas their contents were relatively low (<0.1 and <0.1–0.8 wt%, respectively) in the products (Table S5). The results showed that PFRs were not used as raw materials but might have been introduced through their adherence to other products or dirt. We suggested that only a few PFRs were used in toy products. Despite the lack of regulations for these PFRs in toy products in Japan, several countries, states, or cities have recently regulated certain PFRs (TCEP, TCPP, TDCPP) because of their hazardous health effects [24, 25]. In addition, previous reports show that these regulated PFRs are frequently detected in-house dust and plastic product samples [26] and are associated with allergic diseases and inflammatory reactions [27, 28]. Thus, it is important to focus on the safety management of PFRs used in toy products in Japan.

### Composition of plastic additives based on the year of manufacture

**Fig. 2:**
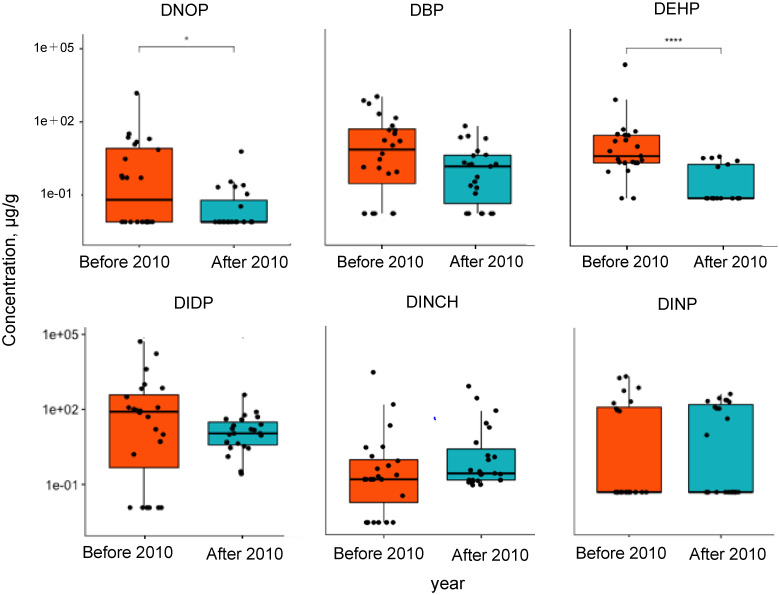
Box-and-whisker plot of concentration of composition and production dates of toys. The median is shown as a thick line, the extent of the box shows the 25th and 75th percentiles and the whiskers show the 5th and 95th percentiles. Comparison of composition and production dates of toys were analyzed using Wilcoxon test, and *p*-values of less than *p* = 0.05 were significant. *: *p* < 0.05, ****: *p* < 0.0001.

**Fig. 3:**
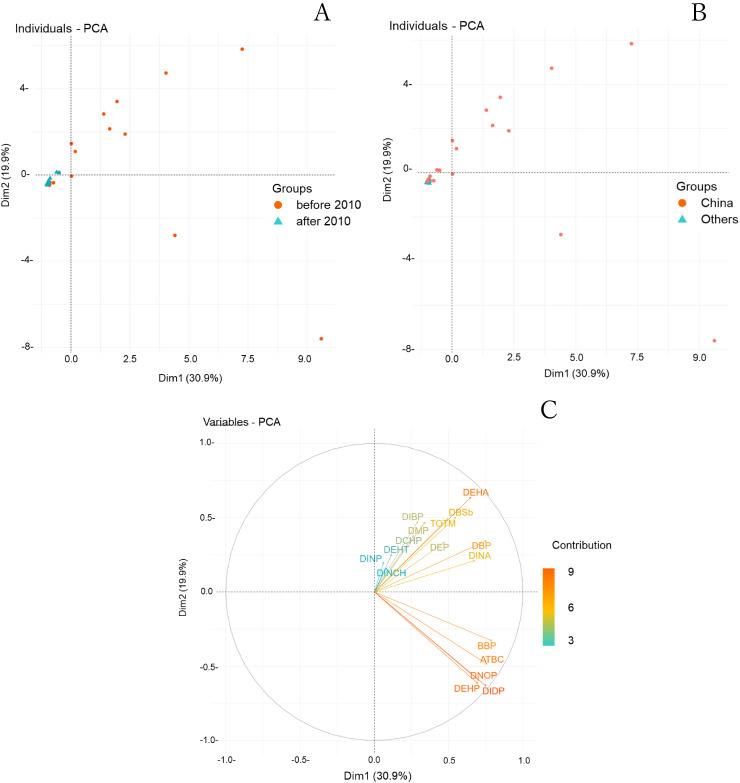
PCA score plot for comparison of year of manufacture (A) and countries of manufacture (B). PCA loading plot from comprehensive analysis of plastic toys by LC-QToFMS/LC-MS/MS (C).

With the expansion of the Food Sanitation Law regulations in 2010 [29], the regulated plasticizers in toy products changed from two PAEs (DEHP and DINP) [30] to six PAEs (DBP, BBP, DEHP, DNOP, DINP, and DIDP). This study evaluated 84 products from various years of manufacture, including designated toys. The toys were classified into two groups: toys manufactured before and after 2010 (Fig. 2). The results indicated that DNOP, DBP, DEHP, and DIDP, as regulated components, were relatively prevalent in products manufactured before 2010. The concentration of DEHP, which has been banned for use in designated toys since 2002, was significantly lower in products manufactured after 2010 due to the regulations. The concentration of DINCH, an alternative ingredient, was relatively high in products manufactured after 2010, although no significant difference was observed. The content rate of plasticizers and other materials in toys manufactured before and after 2010 was also shown in the Table 3. Toys made before 2010 typically had higher levels of regulated substances and also frequently used alternative substances like DEHT and ATBC. For toys made after 2010, most substances were found to be below 0.1%. It’s important to mention that the data in Table 3 only represents a portion of the results for toys with identified manufacturing dates in this study. Furthermore, the PAEs and alternative plasticizers were displayed in a two-dimensional graph (Fig. 3A), and their age distribution was examined. The products manufactured after 2010 were distributed within a small range, whereas the composition of samples manufactured before 2010 exhibited a nonuniform wide distribution. The results indicated that various plasticizers were used as raw materials or additives before 2010, including phthalates, owing to Japan’s limited number of regulated components. These relatively old toys are still readily available at stores of second-hand goods dealers and are widely available locally and internationally. These toys might be significant sources of PAEs exposure for children; therefore, continued investigations for safety control are crucial.

**Table 3:** The content rate of plasticizers and other materials in toys manufactured in different year (wt%).

	**Compounds**	**Before 2010 (n = 24)**			**After 2010 (n = 24)**		
	
		**Minimum**	**Average**	**Maximum**	**Minimum**	**Average**	**Maximum**
Regulatory Compounds	DBP	<0.1	<0.1	0.10	<0.1	<0.1	<0.1
	BBP	<0.1	<0.1	<0.1	<0.1	<0.1	<0.1
	DEHP	<0.1	0.11	2.5	<0.1	<0.1	<0.1
	DNOP	<0.1	<0.1	0.15	<0.1	<0.1	<0.1
	DINP	<0.1	<0.1	0.29	<0.1	<0.1	0.1
	DIDP	<0.1	0.32	5.2	<0.1	<0.1	<0.1

Alternative compounds	DEHT	<0.1	0.12	2.6	<0.1	0.1	1.0
	DIBP	<0.1	<0.1	0.35	<0.1	<0.1	<0.1
	DMP	<0.1	<0.1	<0.1	<0.1	<0.1	<0.1
	DEP	<0.1	<0.1	<0.1	<0.1	<0.1	<0.1
	DCHP	<0.1	<0.1	<0.1	<0.1	<0.1	<0.1
	DEHA	<0.1	<0.1	0.38	<0.1	<0.1	<0.1
	DINA	<0.1	0.12	1.0	<0.1	<0.1	0.1
	ATBC	<0.1	0.32	3.0	<0.1	<0.1	0.1
	DINCH	<0.1	<0.1	0.37	<0.1	<0.1	0.1
	DBSb	<0.1	<0.1	<0.1	<0.1	<0.1	<0.1

<0.1: Content rate of regulatory and alternative compounds was under the regulated value of 0.1%.

Furthermore, when the data of the PAEs obtained from the targeted analysis were overlaid with the country of manufacture, it was observed that most of the Chinese toy samples were plotted within a small range (Fig. 3B). This result was similar to the distribution of the nontargeted and targeted analyses (Fig. 1A). The composition data of each product was expected to be significantly affected after the regulations were revised for the toys and differences in regulatory efforts by each country. In China, the regulations on the content of six PAEs were similar to those of other countries, such as the EU, the USA, and Japan [31]. However, it has been reported that toys imported into Japan containing over 0.1 wt% of DEHP and DBP were voluntarily recalled (2017, 2019) [32, 33]. In such situations, certain countries might not enforce these regulations and safety controls. Chemical substances must be more strictly controlled during manufacture worldwide. Furthermore, countries that import these goods need to inspect and control imports considering their hazards.

### Comparison of concentration of composition and material of toys

The results of the comparison of concentration of composition and material of toys are shown in Fig. S2. From these results, some phthalate esters and alternative plasticizers such as BBP, DBS, DEHA, DEHT, DIBP, DIDP, DMP were relatively high in the toy products made of PET. The concentrations of PAEs and alternatives were particularly high in the finger puppets made of polyethylene terephthalate (PET), despite the lack of use of plasticizers. In addition, PVC products contained relatively high amounts of DEHA, DEHT, DBS, and ATBC, suggesting a trend towards the increased use of alternative components following the regulation of certain phthalate esters. Thus, it is highly probable that the detected phthalates and alternative plasticizers were used for not only PVC products but also other purposes. Although DINP was within the regulation value (0.1 wt%) in the designated toys, its concentration was relatively high in the products of play house toys made in the EU (Denmark). These were very limited data of toys targeted in this study and represent only a portion of the products available domestically and do not reflect information about all toys sold in each country. Regarding the five detected PFR components (TCPP, TPHP, CsDPHP, EHDPP, and TCsP), the highest detection frequency was observed for products made in China. Most of them were hardly detected or existed at low concentrations in products made in Thailand, Japan, and Denmark. Among the other plasticizers in these products, the detection rate of TPHP (15%) was relatively high. The TPHP content was particularly high in the smartphone samples (400 µg/g). Recently, these products have been frequently used by people of all ages, including infants, to watch videos.

Based on above results, although many investigations have focused on PAEs used as plasticizers, mainly in PVC products, products made of other materials might also contain potentially harmful PAEs and other chemical substances. Therefore, future surveys on plastic toy products made of various materials are required, and risk assessments should be conducted for chemicals found in toys and for other daily commodities.

## Conclusion

This study conducted targeted and nontargeted analyses and observed that the chemical profiles of toy products were dependent on the year of manufacture and country of production. Moreover, regulated plasticizers were detected in used products manufactured before regulations were passed, indicating the potential for exposure of infants to these plasticizers. Thus, periodic fact-finding surveys should be conducted for the risk assessment and safety management of domestic toy products. It is necessary to focus on the risk assessment of alternative components, including ATBC, DEHT, and DINA. Moreover, it is important to follow domestic safety management measures and educate nursery school and kindergarten teachers, who frequently come in contact with infants and toddlers, on how to select appropriate toys and avoid exposure to harmful chemicals as much as possible.

## Abbreviations

PAEs: phthalate esters
PFRs: phosphorus flame retardants
LC-QToFMS: high-performance liquid chromatography quadrupole time-of-flight mass spectrometry
LC-MS/MS: highly sensitive and selective liquid chromatography-tandem mass spectrometry
GC-MS: gas chromatography-mass spectrometry
SIM: selected ion monitoring
LOD: limit of detection
LOQ: limit of quantification
SD: standard deviation
PVC: polyvinyl chloride
PE: polyethylene
ABS: acrylonitrile butadiene styrene
TPE: thermoplastic elastomer
MSI: Metabolomics Standards Initiative
MRM: multiple reaction monitoring
PCA: sparse principal component analysis
BFR: brominated flame retardant
DBP: Di-butyl phthalate
BBP: Butyl benzyl phthalate
DEHP: Bis(2-ethylhexyl) phthalate
DNOP: Di-octyl phthalate
DINP: Di-isononyl phthalate
DIDP: Di-isodecyl phthalate
DIBP: Diisobutyl phthalate
DMP: Dimethyl phthalate
DEP: Diethyl phthalate
DCHP: Dicyclohexyl phthalate
DEHA: Bis(2-ethylhexyl)-adipate
DINA: Diisononyl adipate
ATBC: Acetyl tributyl citrate
DINCH: 1,2-Cyclohexane dicaboxylic acid diisononyl ester
DBSb: Dibutyl sebacate
TOTM: Tris(2-ethylhexyl) trimellitate
DBP-*d_4_*: Di-butyl phthalate-*d_4_*
BBP-*d_4_*: Butyl benzyl phthalate-*d_4_*
DMP-*d_4_*: Dimethyl phthalate-*d_4_*
DEP-*d_4_*: Diethyl phthalate-*d_4_*
DEHP-*d_4_*: Bis(2-ethylhexyl) phthalate-*d_4_*
DCHP-*d_4_*: Dicyclohexyl phthalate-*d_4_*
DEHA-*d_8_*: Bis(2-ethylhexyl)-adipate-*d_8_*
DNOP-*d_4_*: Di-octyl phthalate-*d_4_*
DINCH-H6A: 1,2-Cyclohexane dicaboxylic acid diisononyl ester-H6A
TMP: Trimethyl phosphate
TEP: Triethyl phosphate
TCEP: Tris(2-chloroethyl) phosphate
TPP: Tripropyl phosphate
TCPP: Tris(2-chloroisopropyl) phosphate
TDCPP: Tris(1,3-dichloroisopropyl) phosphate
TPHP: Triphenyl phosphate
TIBP: Tris(isobutyl) phosphate
TNBP: Tri-n-butyl phosphate
CsDPhP: Cresyl diphenyl phosphate
TBOEP: Tris(2-butoxyethyl) phosphate
TCsP: Tricresyl phosphate
EHDPP: 2-Ethylhexyldiphenyl phosphate
TCEP-*d_12_*: Tris(2-chloroethyl) phosphate-*d_12_*
TPHP-*d_15_*: Triphenyl phosphate-*d_15_*
TMPP-*d_21_*: Tris(methylphenyl) phosphate-*d_21_*
TEHP-*d_51_*: Tris(2-ethylhexyl) phosphate-*d_51_*

## Supplementary information

**Additional file 1:** **Table S1** Toy sample of this study. **Table S2-1** Analytical condition of nontarget analysis by LC-QToFMS. **Table S2-2** Analytical condition of phthalic esters by LC-MS/MS. **Table S2-3** Analytical condition of phthalate esters by GC-MS (QP2010 plus, Shimadzu). **Table S2-4** Analytical condition of phosphorelated flame retardants by LC-MS/MS. **Table S3-1** MS/MS parameters for the Phthalate esters. **Table S3-2** MS/MS parameters for the Phosphorus flame retardants. **Table S4-1** Calibration curves, detection limits and quantification limits for PAEs and alternative substances with toy sample matrix. **Table S4-2** Calibration curves, detection limits and quantification limits for PFRs with toy sample matrix. **Table S5-1** Nontargeted analysis by LC-QToFMS (1). **Table S5-2** Nontargeted analysis by LC-QToFMS (2). **Table S5-3** Nontargeted analysis by LC-QToFMS (3). **Table S5-4** Nontargeted analysis by LC-QToFMS (4). **Table S5-5** Nontargeted analysis by LC-QToFMS (5). **Table S5-6** Nontargeted analysis by LC-QToFMS (6). **Table S5-7** Nontargeted analysis by LC-QToFMS (7). **Table S5-8** Nontargeted analysis by LC-QToFMS (8). **Table S5-9** Nontargeted analysis by LC-QToFMS (9). **Table S5-10** Nontargeted analysis by LC-QToFMS (10). **Table S6** Concentration of phthalate esters/alternatives and phosphorylated flame retardants in toy samples (ng/g). **Fig. S1-1** Picture of toy sample of this study (No.1∼28). **Fig. S1-2** Picture of toy sample of this study (No.29∼57). **Fig. S1-3** Picture of toy sample of this study (No.58∼84). **Fig. S2** Box-and-whisker plot of comparison of concentration of composition and material of toys (µg/g). The median is shown as a thick line, the extent of the box shows the 25th and 75th percentiles and the whiskers show the 5th and 95th percentiles. Comparison of concentration of composition and material of toys were analyzed using Kruskal-Wallis, and p-values of less than *p* = 0.05 were significant.

## Declarations

### Availability of data and materials

Data used in this study are not open to the public.
