# Supplementary material for: Comprehensive survey on the use of plastic additives in toy products used in Japan
Source: Environ Health Prev Med. 2024 Aug 22;29:43. doi: 10.1265/ehpm.24-00054 (PMC11362670; doi:10.1265/ehpm.24-00054)
Supplement: Supplementary file 1 — Additional file 1: Table S1 Toy sample of this study. Table S2-1 Analytical condition of nontarget analysis by LC-QToFMS. Table S2-2 Analytical condition of phthalic esters by LC-MS/MS. Table S2-3 Analytical condition of phthalate esters by GC-MS (QP2010 plus, Shimadzu). Table S2-4 Analytical condition of phosphorelated flame retardants by LC-MS/MS. Table S3-1 MS/MS parameters for the Phthalate esters. Table S3-2 MS/MS parameters for the Phosphorus flame retardants. Table S4-1 Calibration curves, detection limits and quantification limits for PAEs and alternative substances with toy sample matrix. Table S4-2 Calibration curves, detection limits and quantification limits for PFRs with toy sample matrix. Table S5-1 Nontargeted analysis by LC-QToFMS (1). Table S5-2 Nontargeted analysis by LC-QToFMS (2). Table S5-3 Nontargeted analysis by LC-QToFMS (3). Table S5-4 Nontargeted analysis by LC-QToFMS (4). Table S5-5 Nontargeted analysis by LC-QToFMS (5). Table S5-6 Nontargeted analysis by LC-QToFMS (6). Table S5-7 Nontargeted analysis by LC-QToFMS (7). Table S5-8 Nontargeted analysis by LC-QToFMS (8). Table S5-9 Nontargeted analysis by LC-QToFMS (9). Table S5-10 Nontargeted analysis by LC-QToFMS (10). Table S6 Concentration of phthalate esters/alternatives and phosphorylated flame retardants in toy samples (ng/g). Fig. S1-1 Picture of toy sample of this study (No.1∼28). Fig. S1-2 Picture of toy sample of this study (No.29∼57). Fig. S1-3 Picture of toy sample of this study (No.58∼84). Fig. S2 Box-and-whisker plot of comparison of concentration of composition and material of toys (µg/g). The median is shown as a thick line, the extent of the box shows the 25th and 75th percentiles and the whiskers show the 5th and 95th percentiles. Comparison of concentration of composition and material of toys were analyzed using Kruskal-Wallis, and p-values of less than p = 0.05 were significant. [file ehpm-29-043-s001.docx]

**Comprehensive survey on the use of plastic additives in toy products used in Japan**

Kanae Bekki^1＊^, Akifumi Eguchi^2^, Kohki Takaguchi^2^, Yohei Inaba^1^, Keiko Yukawa^3^, Satomi Yoshida^4^, Kenichi Azuma^5^

^1^ Department of Environmental Health, National Institute of Public Health, Japan

^2^ Center for Preventive Medical Sciences, Chiba University, Japan

^3^ Department of Health Policy and Technology Assessment, National Institute of Public Health, Japan

^4^ Department of Pharmacoepidemiology, Graduate School of Medicine and Public Health, Kyoto University, Japan.

^5^ Department of Environmental Medicine and Behavioral Science, Kindai University Faculty of Medicine, Japan

*Corresponding author phone number: +81-48-458-6258; Fax number: +81-48-458-6270; E-mail: bekki.k.aa@niph.go.jp

Table S1　Toy sample of this study

| No. | Sample | Target age (month) | Material | Rigid/Soft | Made in (country) | Year | Designated/Undesignated toys | New/Used |
| --- | --- | --- | --- | --- | --- | --- | --- | --- |
| 1 | Bathing toy | 36 | PE | Rigid | Japan | Unknown | Designated toy | New |
| 2 | Bathing toy | 36 | PP | Rigid | Japan | 2019 | Designated toy | New |
| 3 | Pacifier | 3 | Unknown | Rigid | China | 2005 | Designated toy | Used |
| 4 | Pacifier | 3 | TPE | Rigid | China | 2018 | Designated toy | Used |
| 5 | Pacifier | 3 | TPE | Soft | Korea | Unknown | Designated toy | New |
| 6 | Pacifier | 3 | ABS | Soft | China | Unknown | Designated toy | New |
| 7 | Pacifier | 3 | Synthetic rubber | Soft | China | 2020 | Designated toy | New |
| 8 | Pacifier | 3 | TPU | Rigid | China | 2018 | Designated toy | New |
| 9 | Pacifier | 0 | Silicone rubber | Soft | China | Unknown | Designated toy | New |
| 10 | Pacifier | 3 | TPE | Soft | Korea | 2019 | Designated toy | New |
| 11 | Pacifier | 6 | TPE | Soft | Thailand | Unknown | Designated toy | New |
| 12 | Block | 24 | PE | Rigid | China | Unknown | Designated toy | New |
| 13 | Block | 36 | PP | Rigid | China | Unknown | Designated toy | New |
| 14 | Block | 18 | ABS | Rigid | Unknown | Unknown | Designated toy | New |
| 15 | Ball | 0 | PU/ABS | Soft | China | Unknown | Designated toy | New |
| 16 | Ball | 18 | PVC | Soft | China | Unknown | Designated toy | New |
| 17 | Ball | 72 | PVC | Soft | China | Unknown | Undesignated toy | New |
| 18 | Ball | 72 | PVC | Soft | China | Unknown | Undesignated toy | New |
| 19 | Play house toys | 36 | PS | Rigid | China | 2018 | Designated toy | Used |
| 20 | Play house toys | 36 | PS | Rigid | China | 2018 | Designated toy | Used |
| 21 | Play house toys | 36 | PS | Rigid | China | 2018 | Designated toy | Used |
| 22 | Play house toys | 36 | PS | Rigid | China | 2018 | Designated toy | Used |
| 23 | Play house toys | 36 | PS | Rigid | China | 2018 | Designated toy | Used |
| 24 | Play house toys | 24 | PE/PP/SBC | Rigid | Denmark | Unknown | Designated toy | New |
| 25 | Play house toys | 24 | PE/PP/SBC | Rigid | Denmark | Unknown | Designated toy | New |
| 26 | Play house toys | 24 | PE/PP/SBC | Rigid | Denmark | Unknown | Designated toy | New |
| 27 | Play house toys | 24 | PE/PP/SBC | Rigid | Denmark | Unknown | Designated toy | New |
| 28 | Play house toys | 24 | PE/PP/SBC | Rigid | Denmark | Unknown | Designated toy | New |
| 29 | Play house toys | 24 | PE/PP/SBC | Rigid | Denmark | Unknown | Designated toy | New |
| 30 | Play house toys | 24 | PE/PP/SBC | Soft | Denmark | Unknown | Designated toy | New |
| 31 | Play house toys | 24 | PE/PP/SBC | Rigid | Denmark | Unknown | Designated toy | New |
| 32 | Play house toys | 24 | PE/PP/SBC | Rigid | Denmark | Unknown | Designated toy | New |
| 33 | Play house toys | 24 | PE/PP/SBC | Rigid | Denmark | Unknown | Designated toy | New |
| 34 | Play house toys | 48 | PE/PP/SBC | Rigid | Denmark | Unknown | Designated toy | New |
| 35 | Play house toys | 36 | PS | Rigid | China | 2018 | Designated toy | New |
| 36 | Play house toys | 36 | PS | Rigid | China | 2018 | Designated toy | New |
| 37 | Play house toys | 36 | PS | Rigid | China | 2018 | Designated toy | New |
| 38 | Play house toys | 36 | PS | Rigid | China | 2018 | Designated toy | New |
| 39 | Play house toys | 36 | PS | Rigid | China | 2018 | Designated toy | New |
| 40 | Play house toys | 36 | PS | Rigid | China | 2018 | Designated toy | New |
| 41 | Play house toys | 36 | PS | Rigid | China | 2018 | Designated toy | New |
| 42 | Toy vehicle | 36 | PS,POM,PU | Rigid | China | 1991 | Designated toy | Used |
| 43 | Toy vehicle | 36 | ABS | Rigid | China | 2014 | Designated toy | Used |
| 44 | Toy vehicle | 36 | ABS | Rigid | China | Unknown | Designated toy | Used |
| 45 | Toy vehicle | 36 | ABS | Rigid | China | Unknown | Designated toy | Used |
| 46 | Toy vehicle | 36 | ABS | Rigid | China | 2004 | Designated toy | Used |
| 47 | Toy vehicle | 36 | ABS | Rigid | Unknown | Unknown | Designated toy | Used |
| 48 | Toy vehicle | 36 | ABS | Rigid | China | 2008 | Designated toy | Used |
| 49 | Toy vehicle | 36 | PP | Rigid | Thailand | 1992 | Designated toy | Used |
| 50 | Toy vehicle | 36 | Unknown | Rigid | Thailand | 1996 | Designated toy | Used |
| 51 | Toy vehicle | 36 | Unknown | Rigid | Thailand | 1996 | Designated toy | Used |
| 52 | Toy vehicle | 18 | ABS/PP/MABS | Rigid | Thailand | 2019 | Designated toy | New |
| 53 | Toy vehicle | 72 | Unknown | Rigid | Unknown | 2004 | Undesignated toy | Used |
| 54 | Toy vehicle | 72 | ABS | Rigid | Taiwan | 2002 | Undesignated toy | Used |
| 55 | Toy vehicle | 72 | ABS | Rigid | China | 2001 | Undesignated toy | Used |
| 56 | Toy vehicle | 72 | ABS | Rigid | China | 2001 | Undesignated toy | Used |
| 57 | Doll | 36 | PVC | Soft | China | 2001 | Designated toy | Used |
| 58 | Doll | 36 | PVC | Soft | China | 2001 | Designated toy | Used |
| 59 | Doll | 36 | PVC | Soft | China | 2004 | Designated toy | Used |
| 60 | Doll | 3 | Rubber | Rigid | China | Unknown | Designated toy | New |
| 61 | Doll | 0 | Rubber | Soft | France | Unknown | Designated toy | New |
| 62 | Doll | 36 | PE/PP | Soft | China | 2019 | Designated toy | Used |
| 63 | Doll | 36 | PE | Soft | China | Unknown | Designated toy | Used |
| 64 | Doll | 36 | PET | Rigid | China | Unknown | Designated toy | Used |
| 65 | Doll | 36 | PET | Soft | China | 1996 | Designated toy | Used |
| 66 | Doll | 36 | PET | Soft | China | Unknown | Designated toy | Used |
| 67 | Doll | 36 | PET | Soft | China | 2000 | Designated toy | Used |
| 68 | Doll | 36 | PET | Soft | China | 1998 | Designated toy | Used |
| 69 | Doll | 36 | PET | Soft | China | 1994 | Designated toy | Used |
| 70 | Doll | 36 | PET | Soft | China | 2000 | Designated toy | Used |
| 71 | Doll | 36 | PET | Soft | China | 2000 | Designated toy | Used |
| 72 | Doll | Unknown | PVC | Soft | China | 2012 | Undesignated toy | Used |
| 73 | Other | 8 | ABS | Rigid | China | 2019 | Designated toy | New |
| 74 | Other | 12 | ABS | Rigid | Unknown | Unknown | Designated toy | New |
| 75 | Other | 6 | PP | Rigid | China | 2020 | Designated toy | New |
| 76 | Other | 0 | PP | Rigid | China | Unknown | Designated toy | New |
| 77 | Other | 72 | PS | Rigid | China | Unknown | Undesignated toy | New |
| 78 | Other | 72 | PS | Rigid | China | Unknown | Undesignated toy | New |
| 79 | Other | Unknown | Silicone rubber | Soft | China | Unknown | Undesignated toy | New |
| 80 | Other | Unknown | Silicone rubber | Soft | Unknown | Unknown | Undesignated toy | New |
| 81 | Other | Unknown | Silicone rubber | Soft | China | Unknown | Undesignated toy | New |
| 82 | Other | 72 | EVA | Soft | China | Unknown | Undesignated toy | New |
| 83 | Other | Unknown | PP/PE | Rigid | Unknown | Unknown | Undesignated toy | New |
| 84 | Other | 72 | PE | Rigid | China | 2007 | Undesignated toy | Used |
| Abbreviations: ABS; acrylonitrile butadiene styrene, EVA; ethylene-vinyl acetate, MABS; methyl methacrylate acrylonitrile butadiene styrene, PE; polyethylene, PET; polyethylene terephthalate, POM; polyoxymethylene, PP; polypropylene, PS; polystyrene, PU; polyurethane, PVC; polyvinyl chloride, SBC; styrenic block copolymer, TPE; thermoplastic elastomer, TPU; thermoplastic polyurethane. | | | | | | | | |

Note: Be aware that the toys targeted in this study represent only a portion of the products available domestically and do not reflect information about all toys sold in the country. Additionally, the toys selected for this study were chosen based on their common use in daily life, referencing manufacturer information, and focusing on those widely used by many people. The manufacturing countries of the products were determined based on the information provided on the toys, resulting in many products being manufactured in China and fewer products from Europe and Japan.

| Table S2-1 Analytical condition of nontarget analysis by LC-QToFMS. |
| --- |
| Instrument: LC-QToFMS (X500R, Sciex) |
| Mobile phase: A) Water 0.1% formic acid, B) Acetonitrile containing 0.1% formic acid |
| Gradient: hold at 5% B for 1 min, 5% to 99% B in 9 min, hold for 0.5 min |
| Flow rate: 0.3 ml/min |
| Column: Ascentis Express C18, 100 mm x 2.1 mm, 2.7 μm (Phenomenex) |
| Column temperature: 40ºC |
| MS setting: 50-800, SWATH mode, positive, negative mode |
| Used library: NIST20, Massbank of North America, Human metabolome database, RIKEN database |

| Table S2-2 Analytical condition of phthalic esters by LC-MS/MS. |
| --- |
| Instrument: LC-MS/MS (Xevo TQ-S, Waters) |
| Mobile phase: A) Water containing 10 mM Ammonium formate, B) Methanol |
| Gradient: hold at 60% B for 0.5 min, 60% to 70% in 3 min, 70% to 95% B in 7.5 min, hold for 3 min, 95% to 60% in 0.5 min |
| Flow rate: 0.3 ml/min |
| Column: Raptor Fluoro Phenyl, 100 mm x 2.1 mm, 1.8 μm (RESTEK) |
| Column temperature: 40ºC |
|  |

| Table S2-3　Analytical condition of phthalate esters by GC-MS (QP2010 plus, Shimadzu) |
| --- |
| GC |
| Analysis Column: Rxi-5ms (30 m×0.25 mm i.d., 0.25 µm) |
| Column oven temp program: 70ºC (2 min)-30ºC/min-200ºC-3ºC/min-250ºC (2.5 min)- 1.5ºC/min-270ºC (5 min) |
| Flow rate: 0.25 ml/min |
| Injection mode: splitless |
| MS |
| Ionization mode : EI |
| Ion source temp : 230ºC |
| IF temperature : 250ºC |
| CID gas : Argon |
| Injection volume : 2 µl |

| Table S2-4 Analytical condition of phosphorelated flame retardants by LC-MS/MS. |
| --- |
| Instrument: LC-MS/MS (Xevo TQ-S, Waters) |
| Mobile phase: A) Water containing 10 mM Ammonium acetate, B) Methanol containing 10 mM Ammonium acetate |
| Gradient: hold at 60% B for 1 min, 60% to 70% in 1 min, 70% to 95% B in 6 min, 95% to 50% in 4 min, hold for 3 min |
| Flow rate: 0.3 ml/min |
| Column: Kinetex C18, 50 mm x 2.1 mm, 1.3 μm (Phenomenex) |
| Column temperature: 50ºC |

| Table S3-1 MS/MS parameters for the Phthalate esters. | | | | | | |
| --- | --- | --- | --- | --- | --- | --- |
| Name | PRABs | Retention time (min) | Precursor ion [M+H]^+^ (m/z) | Quantifier (m/z*)* | Cone (V) | Collision (eV) |
| Di-butyl phthalate | DBP | 5.26 | 279.1 | 149.0 | 12 | 10 |
| Butyl benzyl phthalate | BBP | 5.40 | 313.2 | 149.0 | 16 | 10 |
| Bis(2-ethylhexyl) phthalate | DEHP | 9.41 | 391.3 | 279.0 | 19 | 10 |
| Di-octyl phthalate | DNOP | 9.80 | 391.3 | 261.0 | 18 | 10 |
| Di-isononyl phthalate | DINP | 10.3 | 419.6 | 275.0 | 15 | 10 |
| Di-isodecyl phthalate | DIDP | 10.7 | 447.4 | 149.0 | 15 | 23 |
| Diisobutyl phthalate | DIBP | 5.15 | 279.1 | 149.0 | 12 | 10 |
| Dimethyl phthalate | DMP | 1.68 | 195.1 | 163.1 | 30 | 12 |
| Diethyl phthalate | DEP | 2.48 | 223.2 | 149.1 | 28 | 18 |
| Dicyclohexyl phthalate | DCHP | 6.78 | 331.0 | 167.0 | 15 | 12 |
| Bis(2-ethylhexyl)-adipate | DEHA | 9.53 | 371.3 | 129.0 | 18 | 15 |
| Diisononyl adipate | DINA | 10.28 | 399.8 | 129.0 | 18 | 15 |
| Acetyl tributyl citrate | ATBC | 6.74 | 403.5 | 185.1 | 15 | 12 |
| 1,2-Cyclohexane dicaboxylic acid diisononyl ester | DINCH | 10.54 | 425.7 | 155.2 | 20 | 15 |
| Dibutyl sebacate | DBSb | 7.68 | 315.5 | 139.2 | 18 | 14 |
| Tris(2-ethylhexyl) Trimellitate | TOTM | 11.94 | 547.8 | 305.2 | 18 | 15 |
| Di-butyl phthalate-*d_4_* | DBP*-d_4_* | 5.24 | 283.0 | 209.0 | 12 | 7 |
| Butyl benzyl phthalate-*d_4_* | BBP-*d_4_* | 5.39 | 317.2 | 209.0 | 16 | 5 |
| Dimethyl phthalate-*d_4_* | DMP-*d_4_* | 1.67 | 199.1 | 167.0 | 28 | 10 |
| Diethyl phthalate-*d_4_* | DEP-*d_4_* | 2.47 | 227.2 | 153.1 | 28 | 18 |
| Bis(2-ethylhexyl) phthalate-*d_4_* | DEHP-*d_4_* | 9.41 | 391.3 | 279.0 | 19 | 10 |
| Dicyclohexyl phthalate-*d_4_* | DCHP*-d_4_* | 6.33 | 335.0 | 171.2 | 15 | 12 |
| Bis(2-ethylhexyl)-adipate-*d_8_* | DEHA*-d_8_* | 9.53 | 379.3 | 137.0 | 18 | 15 |
| Di-octyl phthalate-*d_4_* | DNOP-*d_4_* | 9.79 | 395.5 | 153.0 | 18 | 10 |
| 1,2-Cyclohexane dicaboxylic acid diisononyl ester-H6A | DINCH-*H6A* | 10.49 | 431.7 | 161.2 | 19 | 17 |
|  |  |  |  |  |  |  |

| Table S3-2 MS/MS parameters for the Phosphorus flame retardants. | | | | | | |
| --- | --- | --- | --- | --- | --- | --- |
| Name | PRABs | Retention time (min) | Precursor ion [M+H]^+^ (*m/z*) | Quantifier (*m/z*) | Cone (V) | Collision (eV) |
| Trimethyl phosphate | TMP | 0.41 | 141.1 | 109.0 | 30 | 15 |
| Triethyl phosphate | TEP | 0.64 | 183.1 | 99.1 | 30 | 20 |
| Tris(2-chloroethyl) phosphate | TCEP | 0.82 | 287.0 | 99.1 | 25 | 30 |
| Tripropyl phosphate | TPP | 1.83 | 225.1 | 99.1 | 30 | 25 |
| Tris(2-chloroisopropyl) phosphate | TCPP | 1.90 | 327.0 | 99.0 | 30 | 30 |
| Tris(1,3-dichloroisopropyl) phosphate | TDCPP | 2.70 | 430.9 | 99.1 | 30 | 25 |
| Triphenyl phosphate | TPHP | 2.85 | 327.1 | 215.2 | 30 | 30 |
| Tris(isobutyl) phosphate | TIBP | 3.19 | 267.2 | 99.1 | 30 | 30 |
| Tri-*n*-butyl phosphate | TNBP | 3.29 | 267.2 | 99.1 | 30 | 30 |
| Cresyl diphenyl phosphate | CsDPhP | 3.27 | 341.1 | 152.1 | 30 | 30 |
| Tris(2-butoxyethyl) phosphate | TBOEP | 3.76 | 399.2 | 99.1 | 40 | 40 |
| Tricresyl phosphate | TCsP | 4.22 | 369.1 | 243.0 | 30 | 30 |
| 2-Ethylhexyldiphenyl phosphate | EHDPP | 4.77 | 363.2 | 251.0 | 25 | 25 |
| Tris(2-chloroethyl) phosphate-*d_12_* | TCEP-*d_12_* | 0.81 | 299.0 | 67.3 | 35 | 30 |
| Triphenyl phosphate-*d_15_* | TPHP-*d_15_* | 2.80 | 342.2 | 160.1 | 35 | 30 |
| Tris(methylphenyl) phosphate-*d_21_* | TMPP-*d_21_* | 4.16 | 390.1 | 175.1 | 35 | 30 |
| Tris(2-ethylhexyl) phosphate-*d_51_* | TEHP-*d_51_* | 8.41 | 486.6 | 103.0 | 35 | 30 |
|  |  |  |  |  |  |  |

Table S4-1 Calibration curves, detection limits, and quantification limits for PAEs and alternative substances with toy sample matrix.

| Compound | Range (ng/ml) | Regression  equation | coefficient of  determination　(*r^2^)* | LOD (ng/ml) | LOQ (ng/ml) |
| --- | --- | --- | --- | --- | --- |
| DBP | 2-5000 | y = 0.0671x + 3.896 | 0.9948 | 0.1 | 0.1 |
| BBP | 0.1-500 | y = 0.0902x - 0.0243 | 0.9998 | 0.05 | 0.1 |
| DEHP | 5-5000 | y = 0.0135x + 0.1931 | 0.9995 | 1.00 | 5.0 |
| DNOP | 1-2000 | y = 0.0079x - 0.0809 | 0.9988 | 0.50 | 1.0 |
| DINP | 0.2-2000 | y = 0.0136x - 0.2713 | 0.9964 | 0.05 | 0.1 |
| DIDP | 0.1-2000 | y = 0.0201x - 0.5673 | 0.9912 | 0.1 | 0.1 |
| DIBP | 0.5-5000 | y = 0.0613x + 1.9448 | 0.9958 | 0.10 | 0.2 |
| DMP | 0.5-2000 | y = 0.0029x + 0.0517 | 0.9838 | 0.05 | 0.1 |
| DEP | 2-2000 | y = 0.0199x - 0.6056 | 0.9938 | 0.05 | 0.1 |
| DCHP | 0.1-500 | y = 0.0475x - 0.041 | 0.9996 | 0.1 | 0.1 |
| DEHA | 10-2000 | y = 0.0408x + 1.1147 | 0.9931 | 0.05 | 0.1 |
| DINA | 10-5000 | y = 0.0309x - 3.603 | 0.9927 | 0.1 | 0.1 |
| ATBC | 0.2-2000 | y = 0.0283x - 0.4962 | 0.9973 | 0.1 | 0.2 |
| DINCH | 10-5000 | y = 0.0691x - 11.82 | 0.9829 | 5.0 | 10 |
| DBSb | 0.1-2000 | y = 0.0497x - 0.9409 | 0.9958 | 0.05 | 0.1 |
|  |  |  |  |  |  |

Table S4-2 Calibration curves, detection limits and quantification limits for PFRs with toy sample matrix.

|  |  |  |  |  |  |
| --- | --- | --- | --- | --- | --- |
| Compound | Range (ng/ml) | Regression  equation | coefficient of  determination　(*r^2^)* | LOD (ng/ml) | LOQ (ng/ml) |
| TMP | 10-1000 | y = 0.0003x - 0.013 | 0.9868 | 5.0 | 10 |
| TEP | 0.1-1000 | y = 0.0014x + 0.0097 | 0.9912 | 0.05 | 0.1 |
| TPP | 0.1-500 | y = 0.004x + 0.0002 | 0.9990 | 0.05 | 0.1 |
| TCEP | 0.2-500 | y = 0.0015x - 0.0029 | 0.9993 | 0.15 | 0.2 |
| TCPP | 0.1-500 | y = 0.0027x - 0.0042 | 0.9991 | 0.05 | 0.1 |
| TDCPP | 1-100 | y = 0.0228x + 0.0031 | 0.9955 | 0.5 | 2.0 |
| TPHP | 0.1-5000 | y = 0.0033x + 0.0761 | 0.9960 | 0.05 | 0.2 |
| TIBP | 0.2-100 | y = 0.0014x + 0.0014 | 0.9939 | 0.10 | 0.5 |
| TNBP | 0.1-100 | y = 0.0053x + 0.0006 | 0.9977 | 0.05 | 0.2 |
| CsDPHP | 0.1-5000 | y = 0.0009x - 0.0068 | 0.9993 | 0.05 | 0.2 |
| TBOEP | 1-100 | y = 0.0048x + 0.0028 | 0.9942 | 0.5 | 1.5 |
| TCsP | 0.1-500 | y = 0.0024x + 0.0039 | 0.9993 | 0.05 | 0.1 |
| EHDPP | 10-1000 | y = 0.005x + 0.0591 | 0.9949 | 2.0 | 10 |
|  |  |  |  |  |  |

Table 5S-1 Nontargeted analysis by LC-QToFMS (1)


Table 5S-2 Nontargeted analysis by LC-QToFMS (2)

Table 5S-3 Nontargeted analysis by LC-QToFMS (3)

Table 5S-4 Nontargeted analysis by LC-QToFMS (4)

Table 5S-5 Nontargeted analysis by LC-QToFMS (5)

Table 5S-6 Nontargeted analysis by LC-QToFMS (6)

Table 5S-7 Nontargeted analysis by LC-QToFMS (7)

Table 5S-8 Nontargeted analysis by LC-QToFMS (8)

Table 5S-9 Nontargeted analysis by LC-QToFMS (9)

Table 5S-10 Nontargeted analysis by LC-QToFMS (10)

Table S6 Concentration of phthalate esters/alternatives and phosphorylated flame retardants in toy samples (ng/g).

| Compound | Min | Med | (25%, 75%) | Max | Detection rate (%) | Used as raw material (%) |
| --- | --- | --- | --- | --- | --- | --- |
| Phthalate ester/alternative substance | | | | | |  |
| DBP | n.d. | n.d. | ( n.d., 9.8) | 960 | 65 | - |
| BBP | n.d. | n.d. | ( n.d., n.d.) | 12 | 16 | - |
| DEHP | n.d. | n.d. | ( n.d., 3.6) | 25000 | 55 | - |
| DEHT | n.d. | n.d. | ( n.d., 4.0) | 370000 | 40 | 1.1 |
| DNOP | n.d. | n.d. | ( n.d., 0.35) | 1500 | 38 | - |
| DINP | n.d. | n.d. | ( n.d., 167) | 2900 | 48 | - |
| DIDP | n.d. | 11 | (1.7, 73) | 52000 | 88 | 1.1 |
| DIBP | n.d. | 2.3 | ( n.d., 21) | 3500 | 74 | - |
| DMP | n.d. | n.d. | ( n.d., 0.68) | 170 | 31 | - |
| DEP | n.d. | 1.1 | ( n.d., 3.1) | 440 | 63 | - |
| DCHP | n.d. | n.d. | ( n.d., n.d.) | 16 | 5.7 | - |
| DEHA | n.d. | n.d. | ( n.d., 1.4) | 3800 | 47 | - |
| DINA | n.d. | 0.95 | ( n.d., 74) | 11000 | 50 | 3.4 |
| ATBC | n.d. | 0.72 | ( n.d., 37) | 30000 | 52 | 6.8 |
| DINCH | n.d. | 0.20 | (0.14, 0.84) | 3700 | 90 | - |
| DBSb | n.d. | n.d. | ( n.d., 0.15) | 7800 | 39 | 1.1 |
| Phosphorylated flame retardant | | | | | |  |
| TMP | n.d. | n.d. | ( n.d, n.d.) | n.d. | n.d. |  |
| TEP | n.d. | n.d. | ( n.d, n.d.) | n.d. | n.d. |  |
| TPP | n.d. | n.d. | ( n.d, n.d.) | n.d. | n.d. |  |
| TCEP | n.d. | n.d. | ( n.d, n.d.) | n.d. | n.d. |  |
| TCPP | 0.75 | 0.75 | (0.75, 0.75) | 0.75 | 1.1 |  |
| TDCPP | n.d. | n.d. | ( n.d, n.d.) | n.d. | n.d. |  |
| TPHP | 1.2 | 73 | (18, 140) | 402 | 15 |  |
| TIBP | n.d. | n.d. | ( n.d, n.d.) | n.d. | n.d. |  |
| TNBP | n.d. | n.d. | ( n.d, n.d.) | n.d. | n.d. |  |
| CsDPHP | 0.66 | 1.2 | (0.66, n.d.) | 1.7 | 2.3 |  |
| TBOEP | n.d. | n.d. | ( n.d, n.d.) | n.d. | n.d. |  |
| TCsP | 0.21 | 5.0 | (1.1, 7.6) | 15 | 14 |  |
| EHDPP | 20 | 52 | (20, n.d.) | 83 | 2.3 |  |
| n.d. = not determines. | | | | | |  |


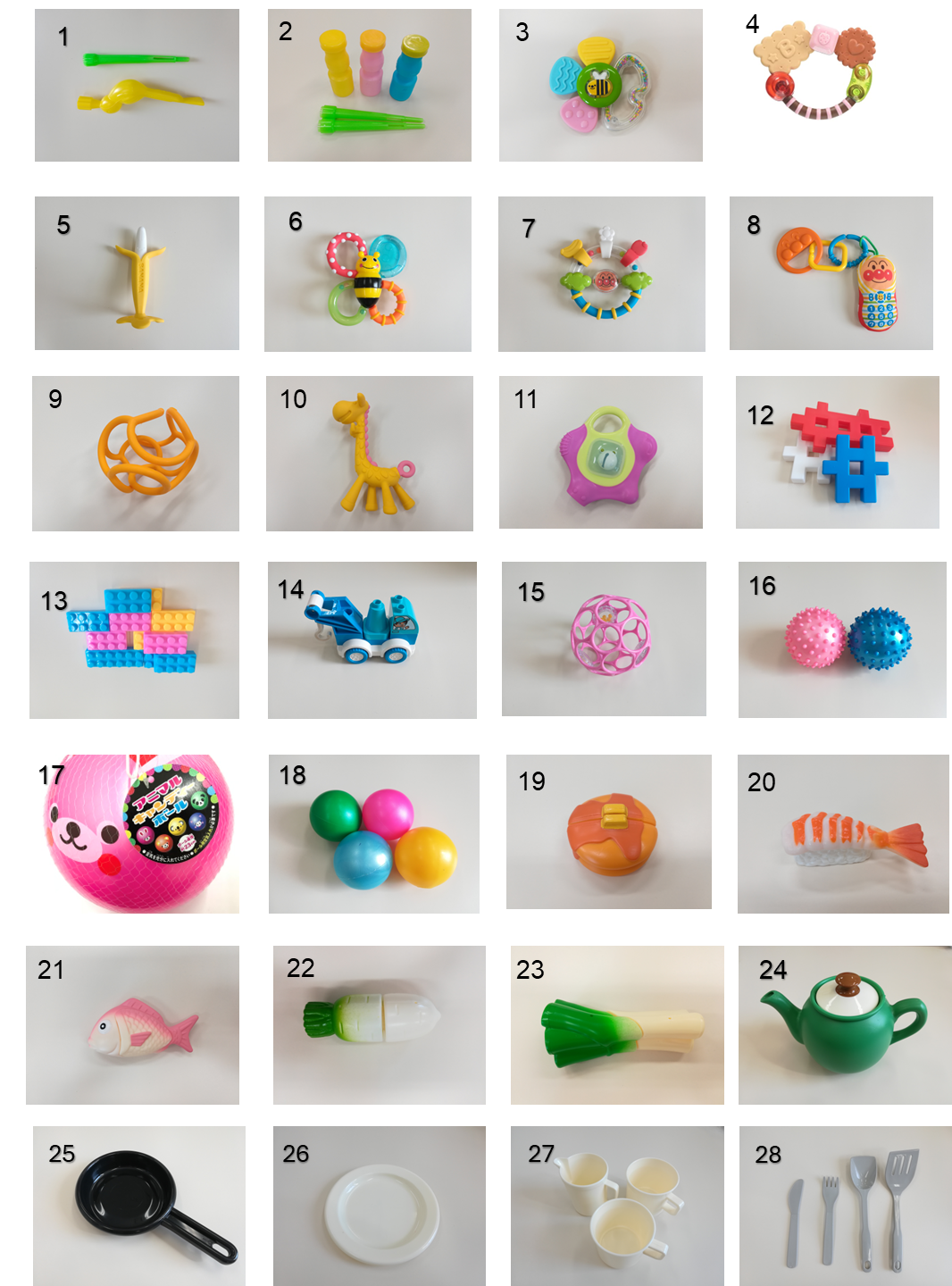


Figure S1-1 Picture of toy sample of this study (No..1~28).


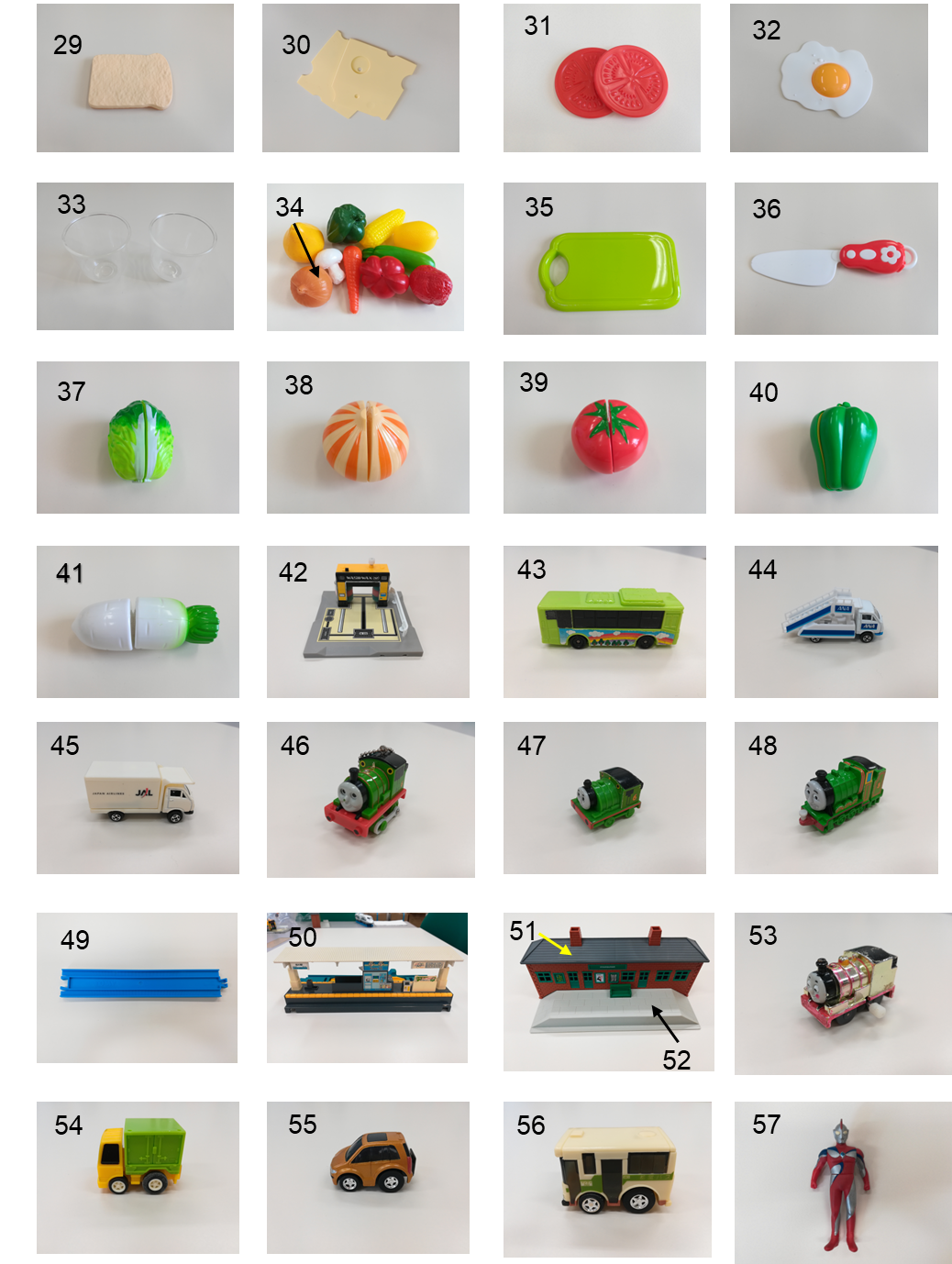


Figure S1-2 Picture of toy sample of this study (No..29~57).


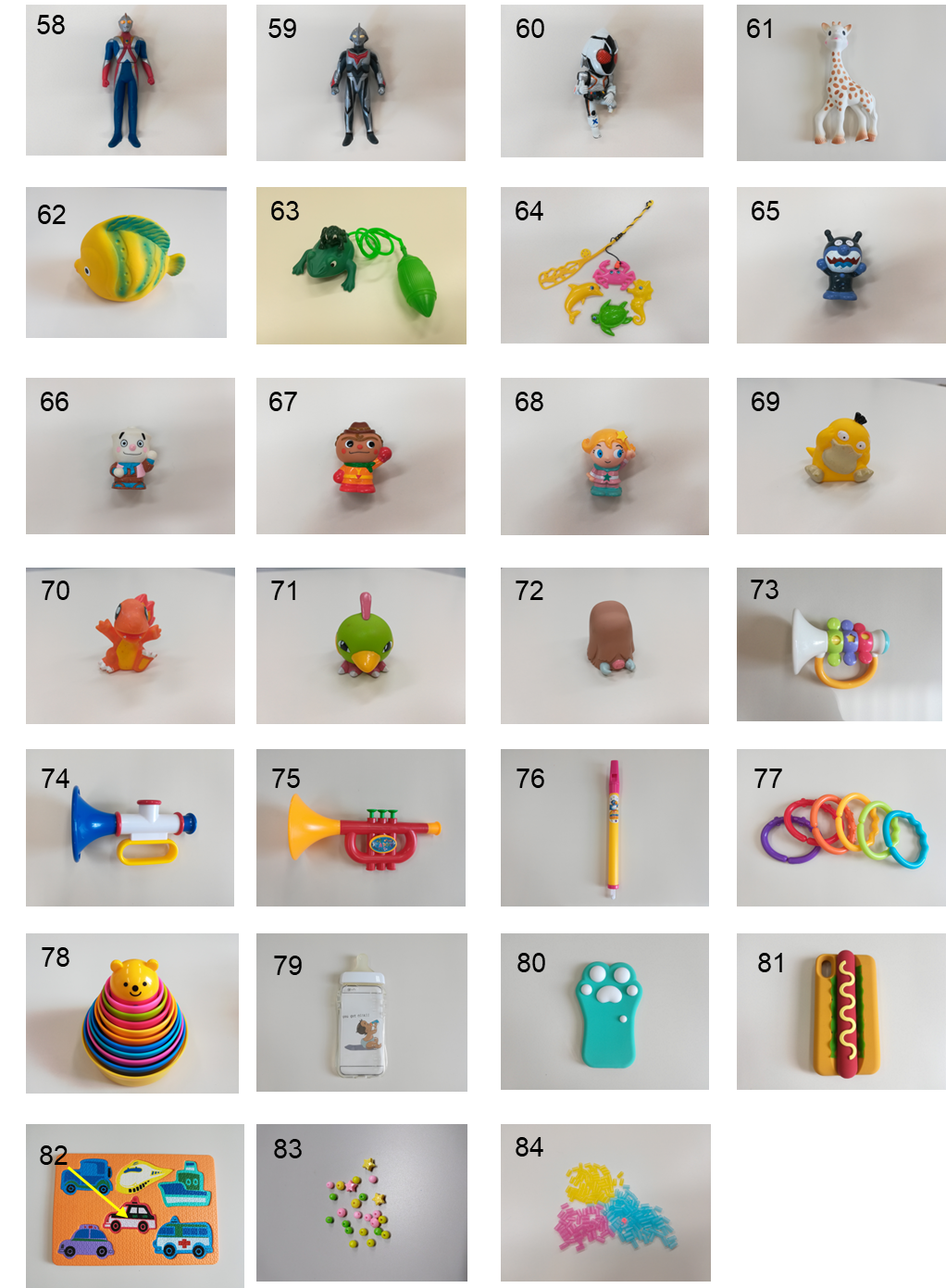


Figure S1-3 Picture of toy sample of this study (No..58~84).


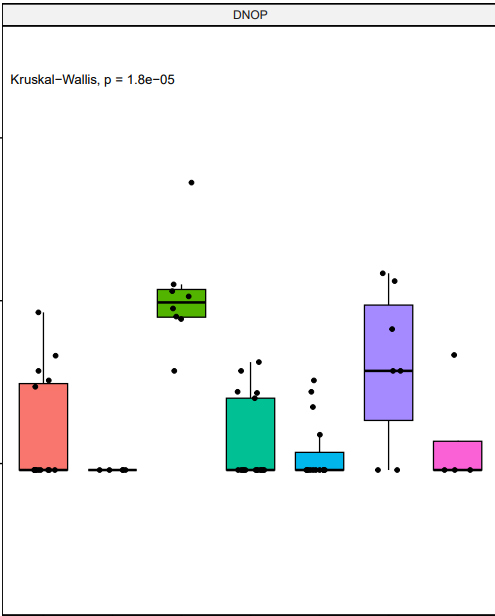

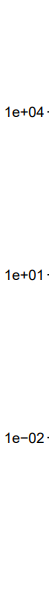


DNOP, p=1.8e-05


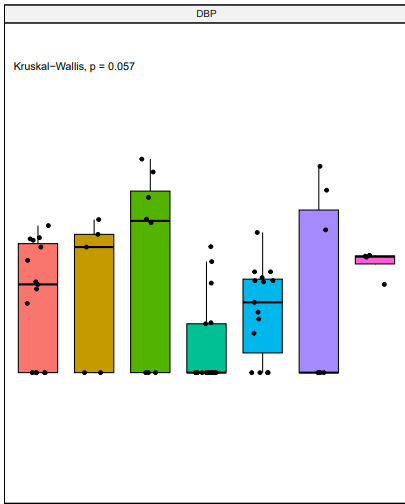


DBP, p=0.057


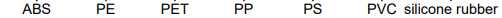

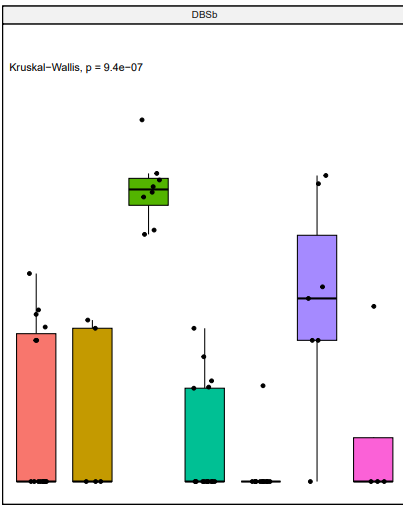


DBSb, p=9.4e-07


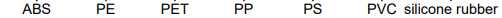

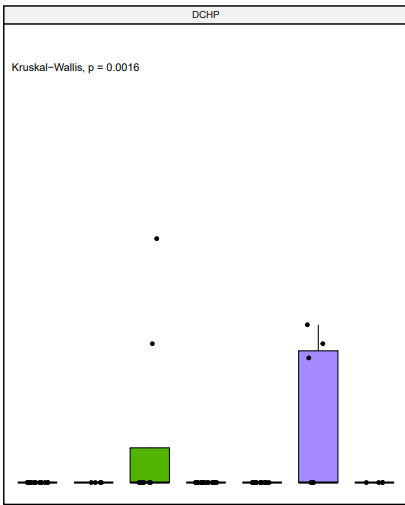


DCHP, p=0.0016


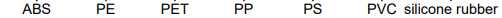

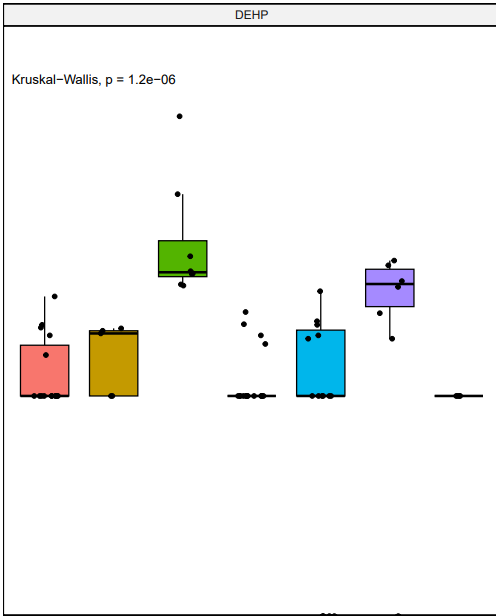

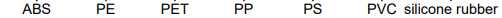


DEHP, p=1.2e-06


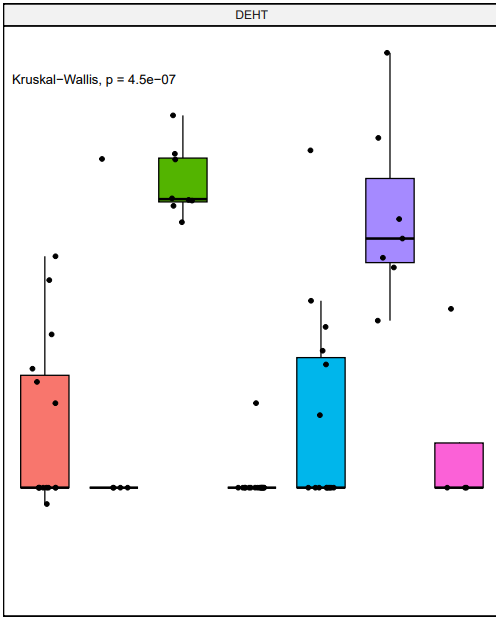


DEHT, p=4.5e-07


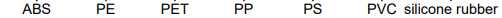

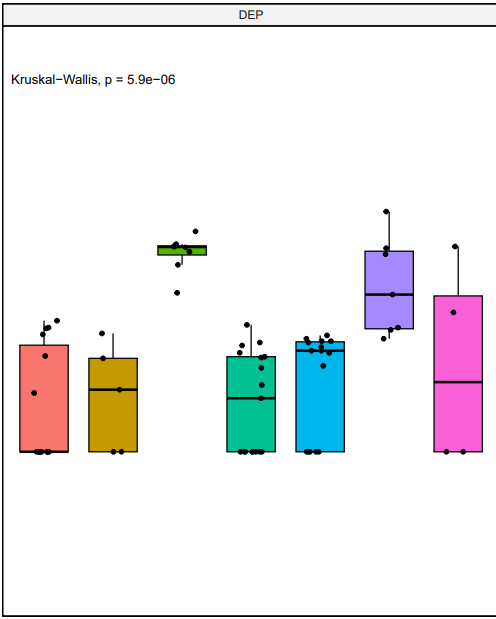


DEP, p=5.9e-06


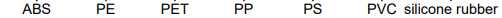

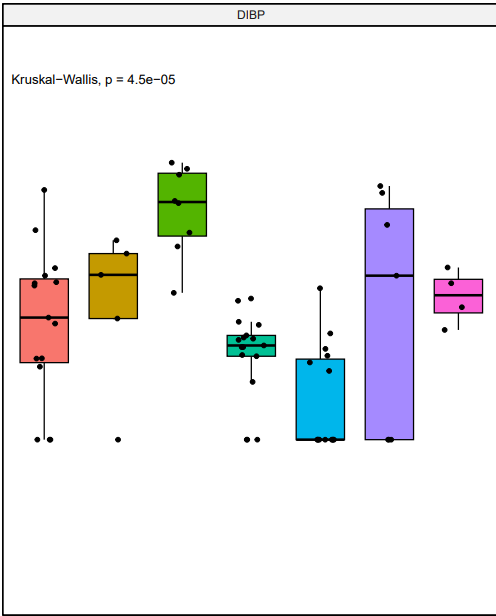


DIBP, p=4.5e-05


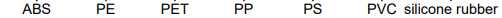

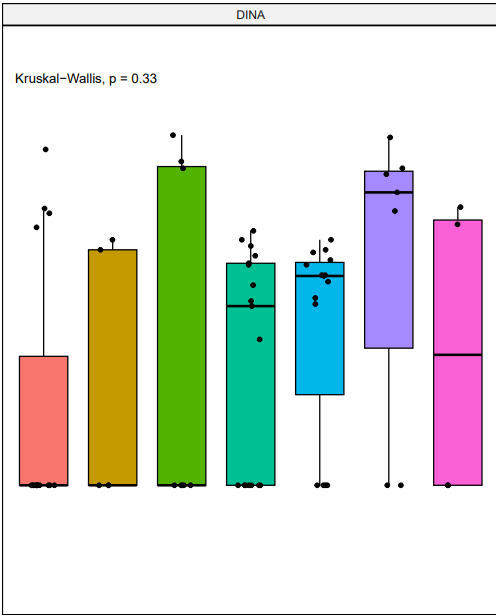


DINA, p=0.33


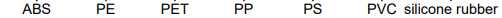

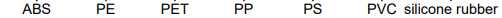

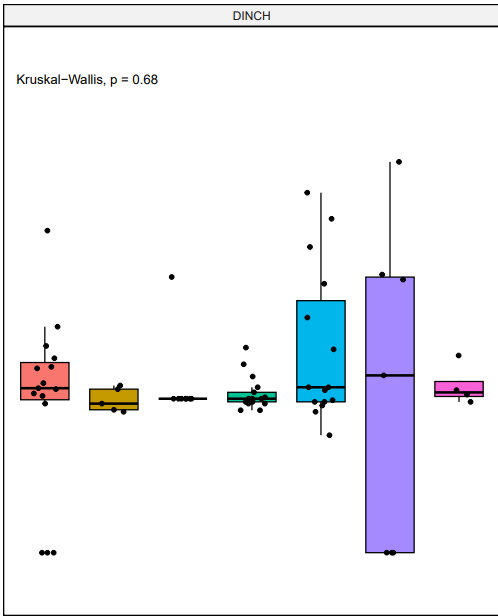


DINCH, p=0.68


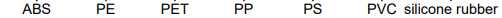

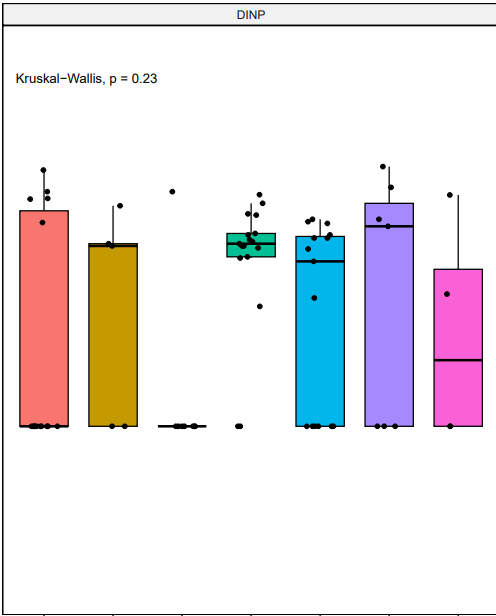


DINP, p=0.23


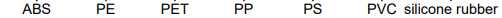

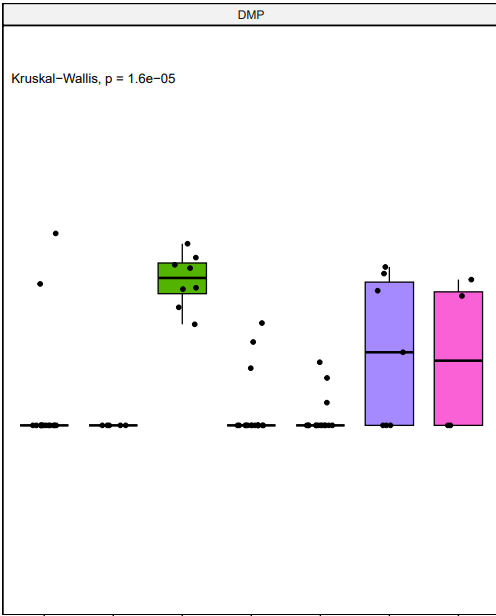


DMP, p=1.6e-05


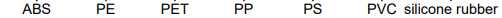

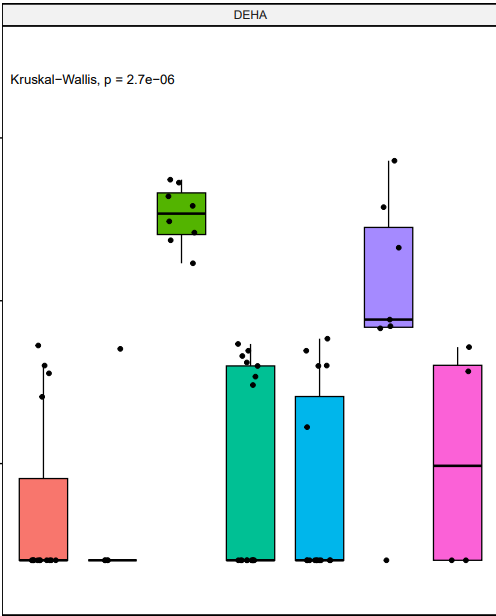


DEHA, p=2.7e-06


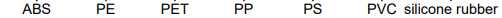

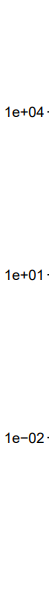

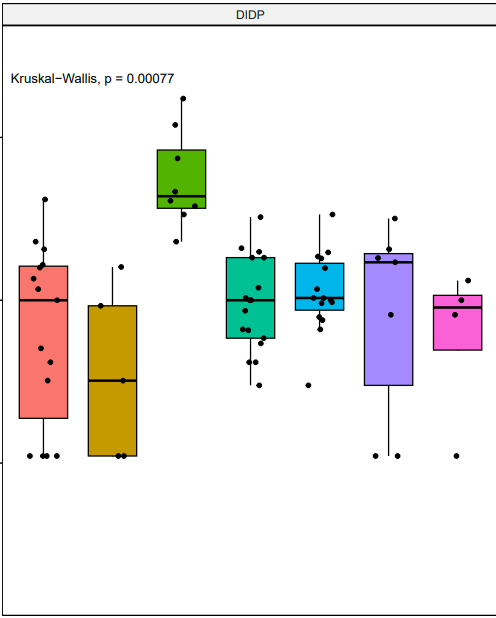


DIDP, p=0.00077


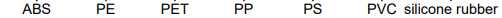

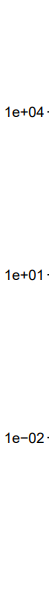

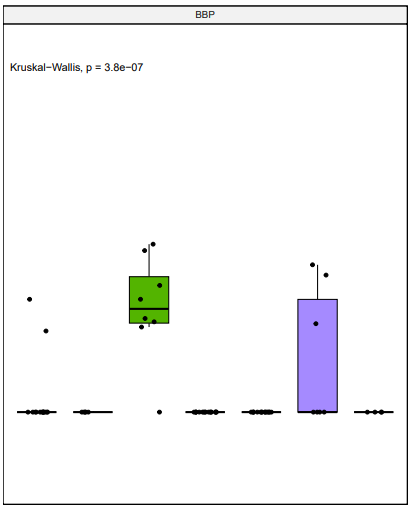


BBP, p=3.8e-07


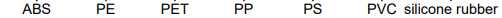

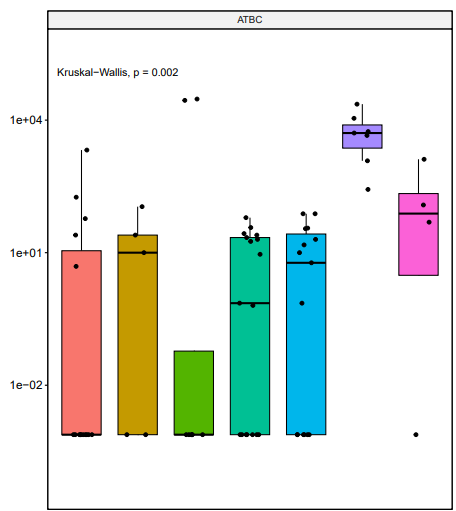


ATBC, p=0.002


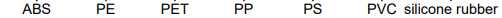


phthalate esters/alternatives, µg/g

Figure S2 Box-and-whisker plot of comparison of concentration of composition and material of toys (µg/g). The median is shown as a thick line, the extent of the box shows the 25th and 75th percentiles and the whiskers show the 5th and 95th percentiles. Comparison of concentration of composition and material of toys were analyzed using Kruskal-Wallis, and *p*-values of less than *p* = 0.05 were significant.
